# Cancer Chemoprevention by Resveratrol: The p53 Tumor Suppressor Protein as a Promising Molecular Target

**DOI:** 10.3390/molecules22061014

**Published:** 2017-06-18

**Authors:** Danielly C. Ferraz da Costa, Eliane Fialho, Jerson L. Silva

**Affiliations:** 1 Instituto de Nutrição, Universidade do Estado do Rio de Janeiro, Rio de Janeiro, 20550-013, Brazil; danielly.costa@uerj.br; 2 Instituto de Nutrição Josué de Castro, Universidade Federal do Rio de Janeiro, Rio de Janeiro, Brazil; 3 Instituto de Bioquímica Médica Leopoldo de Meis & Instituto Nacional de Ciência e Tecnologia de Biologia Estrutural e Bioimagem, Universidade Federal do Rio de Janeiro, Rio de Janeiro, RJ, 21941-902, Brazil

**Keywords:** resveratrol, cancer, signal transduction, p53, clinical trials

## Abstract

Increasing epidemiological and experimental evidence has demonstrated an inverse relationship between the consumption of plant foods and the incidence of chronic diseases, including cancer. Microcomponents that are naturally present in such foods, especially polyphenols, are responsible for the benefits to human health. Resveratrol is a diet-derived cancer chemopreventive agent with high therapeutic potential, as demonstrated by different authors. The aim of this review is to collect and present recent evidence from the literature regarding resveratrol and its effects on cancer prevention, molecular signaling (especially regarding the involvement of p53 protein), and therapeutic perspectives with an emphasis on clinical trial results to date.

## 1. Introduction

Cancer is a source of significant and growing mortality worldwide, with an increase to 19.3 million new cancer cases per year projected for 2025. More than half of cancer cases and mortality occur in low- and middle-income countries [1,2]. Current treatments for cancer include surgery, radiotherapy and systemic treatments comprising cytotoxic chemotherapy, hormonal therapy, immunotherapy, and targeted therapies [3].

Epidemiologic and experimental studies have suggested favorable effects of dietary polyphenols through their anti-carcinogenic properties. Resveratrol represents a group of diet-derived cancer chemopreventive agents encompassing, among others, curcumin, tea polyphenols, probiotics, and lycopene, which have attracted great interest in the cancer chemoprevention community because of their ability to minimally engage this process. Thus, these agents have been explored as potential therapeutic agents in humans [4].

The aim of this review is to collect and present recent evidence in the literature regarding resveratrol and its effects on cancer prevention, molecular signaling (especially regarding the involvement of the p53 protein), and therapeutic perspectives, with an emphasis on clinical trial results.

## 2. Cell Signaling Pathways Regulated by Resveratrol in Cancer

Resveratrol (3,4′,5-trihydroxy-trans-stilbene) is a naturally occurring polyphenol that is found at low concentrations in more than 70 plant species, including grapes, cranberries and peanuts, as well as in a number of herbal remedies [5]. Resveratrol from grapes is efficiently extracted during the wine-making process, and it has been speculated that red wine, in particular, may be the most important dietary source of this microcomponent [6].

In PubMed (http://www.ncbi.nlm.nih.gov/pubmed/), a search for “Resveratrol and Cancer” generated 2325 hits (April 2017). Among its wide range of biological activities, resveratrol has attracted considerable attention due to its role in regulating multiple signal transduction pathways involved in carcinogenesis. Since 1997, when Jang et al. [7] first reported the in vivo antitumor properties of resveratrol, accumulating data have demonstrated its ability to modulate a number of intracellular mediators in cancer initiation, promotion and progression. Therefore, many chemopreventive and chemotherapeutic mechanisms to prevent, arrest, or delay tumor development by this microcomponent have been proposed [5,8,9,10].

Resveratrol regulates a variety of processes and signaling pathways that involves procarcinogen bioactivation and carcinogen detoxification [11,12]; reduction of oxidative stress [13] and inflammation [14]; apoptosis induction through the activation of both extrinsic and intrinsic pathways [15,16,17,18,19,20]; and other anticancer effects [21,22]. Resveratrol affects the three phases of carcinogenesis, tumor initiation, promotion and progression, and it also suppresses the final steps of carcinogenesis, e.g., angiogenesis and metastasis [11,23]. Resveratrol also impacts mitochondrial functions (the respiratory chain, oncoproteins, gene expression, among others), including those directly involving the p53 protein tumor suppressor protein [24]. Finally, in various cancer types, resveratrol behaves as a chemosensitizer that lowers the threshold of cell death induction by classical anticancer agents and counteracts tumor cell chemoresistance [25,26].

In vitro and in vivo studies have shown that resveratrol can act as a tumor-initiation suppressor by modulating phase I and phase II cytochrome P450 enzymes (CYPs) [5,12,27,28,29,30]. Resveratrol blocks the transcriptional activation of CYPs and inhibits the activity of CYP1A1, CYP1B1 and CYP1A2, which are phase I enzymes responsible for activating xenobiotics, thus halting the transformation of procarcinogen agents into potential carcinogens [12,27,28]. Additionally, resveratrol enhances the expression and/or activity of phase II enzymes, including glutathione peroxidase, glutathione S-transferase, UDP-glucuronyltransferase, NAD(P)H:quinone oxidoreductase, and heme oxygenase, among others [5,29,31], subsequently stimulating carcinogen detoxification processes.

The role of resveratrol in the intracellular redox status has been described in many studies. Like other polyphenols, this microcomponent acts as an important cellular antioxidant, and its effects are dependent on the concentration tested and the cell type used. However, it has been proposed that resveratrol may also act as a pro-oxidant agent, representing one of the antineoplastic mechanisms of action involved in tumor cell death. Likewise, resveratrol has been shown to decrease the mitochondrial membrane potential and increase reactive oxygen species (ROS) generation, thus promoting apoptosis [32,33].

Some effects of resveratrol have been attributed to its estrogenic activity resulting from its structural similarity to diethylstilbestrol, a synthetic estrogen. Resveratrol can bind to estrogen receptors and may function as an agonist or antagonist, leading to opposite responses depending on the concentration, competition and expression of estrogen receptors in the cells. In MCF-7 human breast cancer cells, for example, which express these receptors, resveratrol may act as a superagonist [34].

Resveratrol is recognized as a potent tumor growth inhibitor in diverse experimental models. Previous studies have established its anticancer effects in a variety of cultured tumor cell lines, including colon [35,36,37], breast [19,38,39], prostate [40,41], pancreatic [42,43,44], lung [19,45,46], melanoma [47,48], glioma [49,50], and leukemia cells [51,52], among others. It has been suggested that the anti-proliferative properties of this microcomponent are related to its capacity to block DNA synthesis and to interfere with various stages of cell progression by regulating the machinery of proteins involved in cell cycle control. Hsieh and colleagues [53] first reported the ability of resveratrol to control growth and cell cycle transitions in human breast carcinoma cell lines with different metastatic potentials: highly invasive MDA-MB-435 and the minimally invasive MCF-7 cells. Resveratrol exerts a greater inhibitory effect on MDA-MB-435 cells, in which a reduction of the fraction of cells in the G1 phase and a corresponding accumulation of cells in S phase was observed. Since then, many other studies have reported that resveratrol at micromolar concentrations arrests the cell cycle of a variety of human cancer cell lines at the G1/S transition, in S phase or in the G2/M phase [5,54,55]. Although several studies have found that the induction of cell cycle arrest by resveratrol is a reversible process and does not trigger apoptosis, many other authors have reported that this process is often followed by apoptotic cell death [56].

Investigations regarding the effects of resveratrol on apoptosis induction in tumor cells have revealed that this compound can stimulate cell death by modulating proteins involved in both the intrinsic and extrinsic apoptotic pathways [56]. Numerous lines of evidence suggest that resveratrol affects the activation of pro-apoptotic and the inhibition of anti-apoptotic molecules. These mechanisms have been described in culture cells and in vivo in tumor models that have been chemically induced in animals and xenographic models in nude mice [55]. Studies suggest that resveratrol-mediated apoptosis includes the activation of death receptors such as Fas and TRAIL; the activation of Bax, Bak, PUMA, Noxa and Bim; and the inhibition of Bcl-2 and Bcl-XL. Some of these responses are mediated by the activation of the p53 tumor suppressor protein by resveratrol, as will be later discussed.

In addition, resveratrol demonstrates important anti-angiogenic effects, thus contributing to the reduction of the metastatic potential of tumor cells. The following mechanisms are involved in this process: the inhibition of extracellular matrix metalloproteinases gene expression, such as MMP-2 and MMP-9, which are involved in tumor invasiveness; and the inhibition of the expression of HIF-1α and VEGF, factors that are directly related to new blood vessel formation [54,55].

Although some of the anti-carcinogenic effects of resveratrol have already been shown to be triggered by the activation of extracellular receptors [57], there is evidence that its internalization by cells is necessary for the activation of some specific intracellular targets. It has been demonstrated by multiphoton microscopy (two-photon excitation) that resveratrol, but not its glycosylated or sulfated metabolites, is efficiently captured by neuroblastoma cells, allowing this molecule to exert its antitumor effects, unlike metabolites [58]. Additionally, the importance of cell membrane microdomains in the early biochemical events triggered by resveratrol resulting in cancer cell death, as well as in its absorption and distribution, has been previously described. Resveratrol accumulates in lipid rafts and is then taken up by cells through raft-dependent endocytosis. These events allow for the activation of kinase pathways and redistribution of cell death receptors within lipid microdomains, ultimately leading to apoptotic cell death [17].

Although diverse in vitro and cell culture experiments have described the pro-apoptotic potential of resveratrol, a study conducted in a xenograft model has shown that this compound can inhibit tumor growth in vivo when administered orally, but there is no evidence of apoptosis induction. This finding may be due to the low bioavailability of resveratrol in animals. However, the tumor growth inhibition can be explained by other mechanisms that are independent of a pro-apoptotic effect, such as the anti-proliferative and anti-angiogenic activity of resveratrol [59]. The systemic administration of resveratrol inhibits the initiation and growth of tumors in a variety of cancer models in rodents. The efficacy of low daily doses of resveratrol (200 μg/kg body weight, for example) in animals with induced colon carcinogenesis suggests that even low concentrations of the compound, such as those obtained by the ingestion of red wine, could be therapeutic in some cases. However, the protective effects of resveratrol are observed more frequently when higher and pharmacologically achievable concentrations are used [60]. There are also studies showing that resveratrol, when administered in animals through peritumoral and intratumoral injections, presents a bioavailability in plasma and tumor significantly higher than that found after oral administration, thus improving the responses of tumor cells to this compound [58].

In addition, studies regarding the effects of resveratrol on in vivo models of spontaneous carcinogenesis are limited and contradictory. Resveratrol supplementation in these experimental models has shown positive, neutral as well as negative outcomes, depending on resveratrol route of administration, dose, tumor model, species, and molecular properties intrinsic to the cancer cell type as already described by others [21,61,62].

Because of these pleiotropic effects, scientists consider resveratrol to have potential as an anticancer drug, and efforts have focused on obtaining a thorough understanding of its mechanisms of action.

## 3. The p53 Tumor Suppressor Protein as a Chemotherapeutic Target of Resveratrol

p53 is a critical tumor suppressor protein that has an essential role in cancer prevention. Wild-type p53 blocks tumor development through the induction of cell cycle arrest and/or apoptosis. As a transcription factor, p53 regulates the transcription of specific target genes involved in these processes, thus triggering growth arrest, senescence, cell differentiation and/or cell death under different stress conditions, such as DNA damage, oncogene activation, hypoxia, and telomere erosion, among others [63,64,65].

The p53 pathway is extremely sensitive to small amounts of DNA molecule damage, which is crucial for the early detection of genetic lesions in tumors [66]. In response to this damage, the activity of specific proteins that are responsible for p53 activation is stimulated, such as checkpoint 2 protein (Chk2). Chk2 is a serine/threonine kinase and, when phosphorylated by ATM, may activate p53, thus promoting its stabilization through phosphorylation at the serine 20 residue, which prevents MDM2-mediated p53 degradation [67]. Consequently, p53 stabilization leads to the activation of *ci*p*1*, one of its main target genes. The *ci*p*1* product is the p21 protein, which acts as an inhibitor of cyclin-dependent kinases (CDKs) in G1 phase of the cell cycle. CDKs allow the transition from G1 to S phase and from G2 to M phase, thus promoting the synthesis and replication of DNA and cell division. Inhibition occurs by preventing the phosphorylation of several regulatory proteins, blocking cell cycle progression [68].

p53 also modulates cell death pathways by mechanisms that are dependent or independent of its activity as a transcription factor. p53-mediated apoptosis occurs through the induction of the transcription of its target gene, whereas p53-independent apoptosis occurs mainly by the interaction of p53 with anti-apoptotic or pro-apoptotic proteins. Numerous studies have shown that p53 can induce the expression of proteins involved in both intrinsic and extrinsic apoptotic pathways [69]. Some mitochondrial proteins show increased expression in response to p53, such as NOXA, PUMA and p53AIP1. Additionally, p53 stimulates the transcription of members of the pro-apoptotic Bcl2 gene family, such as Bax and Bak, allowing the release of cytochrome c into the cytoplasm and their binding to protease activating factor apoptotic (Apaf-1), which leads to the oligomerization of Apaf-1 complex/caspase 9 (apoptosome). This complex recruits pro-caspase 9 and activates effector caspases. p53 can also promote apoptosis through the activation of death receptors, including Fas, DR4 and DR5 [70].

The ability of wild-type p53 overexpression to induce apoptosis may be a major reason why cancer cells frequently exhibit disabled p53 or p53-mediated pathways during the oncogenic process. The same genetic changes that cause the loss of apoptosis during tumor development may also result in tumor chemoresistance to anticancer therapies that kill tumor cells by apoptosis. Elucidation of the mechanisms involved in these cellular responses may provide insights into strategies to induce cell death and suggest new targets for improving cancer treatment.

Consistent evidence suggests that resveratrol can induce p53-dependent cell death in a variety of cancer cell lines [19,71,72,73,74,75]. Previous studies have demonstrated that this microcomponent promotes the activation and stabilization of cellular levels of p53 protein in tumor cell cultures by inducing post-translational modifications, such as phosphorylation and acetylation. Such modifications are required for the transcriptional activation of p53-responsive genes [76]. Interestingly, resveratrol and other polyphenolic compounds can also trigger apoptosis independently of the p53 cellular status. Alternative mechanisms, including the role of p73, a p53-related tumor suppressor, have been reported in recent studies [77].

Resveratrol-induced p53 activation and apoptosis pathways have been shown to be mediated by MAP kinases (mitogen-activated protein kinases). She and colleagues [72] demonstrated for the first time that resveratrol could increase endogenous levels of p53, especially in the phosphorylated state, in epidermal JB6 cells, which constitute a well-developed model of cell culture to study tumor progression. Likewise, levels of phosphorylated protein kinases (ERKs, p38 kinase and JNKs) increase in the presence of resveratrol in a time-dependent manner [73]. In MCF-7 human breast cancer cells, a mechanism has been suggested in which resveratrol, when binding to an integrin located in the plasma membrane, triggers the activation of ERKs, which in turn phosphorylate p53 protein [57].

In MCF-7 cells, the mechanisms by which resveratrol induces cell death appear to involve the activation of MAP kinases (MAPK and ERKs 1 and 2), which is associated with the phosphorylation and acetylation of p53 at serine residues [78]. Other authors have demonstrated that in these cells, resveratrol treatment resulted in a dose-dependent inhibition of cell growth and an accumulation of cells in S phase of the cell cycle. In addition, the anti-proliferative effects of resveratrol are associated with inhibition of cyclin D and CDKs and induction of p53 and the inhibitor of CDKs, p21. In these cells, resveratrol-induced apoptosis involves the activation of caspase 9, reduced expression of the anti-apoptotic proteins Bcl-2 and Bcl-xL and increased levels of the pro-apoptotic Bax protein [79].

Another study identified the role of p53-dependent and -independent pathways in resveratrol-induced apoptosis in breast cancer cells and showed that this compound could induce cell death in cells expressing wild-type p53 but not in those expressing a mutant form of the protein [71]. In cancer cells derived from lung (A549), liver (HepG2), thyroid (FTC 236 and FTC 238), and osteosarcoma cells (SJSA1), among others, the anti-proliferative and pro-apoptotic effects of resveratrol have been shown to be mediated by p53 [80,81,82]. In prostate cancer cells, resveratrol increased the expression of p53-p(ser15) and/or p53-ac(lys382) and total p53 protein without a change in p53 mRNA. This compound also induced p53 translocation to mitochondria and promoted cell cycle alterations, as well as the induction of apoptosis [83,84,85,86,87].

A study by our group highlighted that, despite the p53-independent apoptosis reported for some cancers, a functional wild-type p53 is required to increase the sensitivity of tumor cells to resveratrol. We showed that transient transfection of a wild-type p53 gene caused H1299 lung cancer cells (p53^−/−^) to become more responsive to the pro-apoptotic properties of resveratrol, similar to the findings in p53-positive MCF-7 cells. These findings suggest a possible therapeutic strategy based on the use of resveratrol for the treatment of tumors that are typically unresponsive to conventional therapies due to the loss of normal p53 function [19].

In the last 15 years, our group has also evaluated the stability and aggregation properties of wild-type and mutant p53. We have previously demonstrated the formation of different types of aggregates after the physical induction of p53 core domain (p53C) unfolding [88] and the ability of a small cognate double-stranded DNA to stabilize both p53C and full-length p53, thus rescuing aggregated and misfolded species of the protein [89]. In addition to these in vitro studies, we used co-localization assays to detect p53 aggregates in archived samples of breast cancer tissues expressing the p53 mutant R248Q and other p53 hot-spot mutants [90]. We have also shown that hot-spot mutants have a greater tendency to aggregate than wild-type p53. Using different techniques, we demonstrated the amyloid nature of the aggregates. It has been demonstrated that seeding p53C R248Q mutant oligomers and fibrils triggers the aggregation of wild-type p53, a behavior typical of a prion. We also observed the co-localization of full-length p53 and aggregates in breast cancer cell lines. In MDA-MB 231 cells, R280K p53 mutant expression revealed a massive accumulation of p53 aggregates in the cell nucleus [91]. Co-aggregation of mutant p53 with other proteins has also been described and may lead to a gain-of-function phenotype. Mutant p53 aggregation appears to occur together with its paralogs p63 and p73 [92,93,94]. Amyloid aggregates of mutant p53 have also been discovered in other types of malignant tumors, such as malignant skin tumors [95] and ovarian cancer [96]. These findings may reveal the biological significance of the prion-like behavior of oncogenic p53 mutants and help with the development of new strategies to disrupt the formation of aggregates [91,97,98,99].

Resveratrol has been shown to inhibit amyloid aggregation by binding to several amyloidogenic proteins such as transthyretin [100,101,102], the islet amyloid polypeptide (IAPP) [103], amyloid beta peptide [104] and alpha-synuclein [105]. Our group has studied the interaction of resveratrol with p53 and evaluated its effect on p53 amyloid aggregation [106,107]. The results have shown that part of the anti-tumoral effects of resveratrol might be related to the inhibition of p53 aggregation.

**Figure 1:**
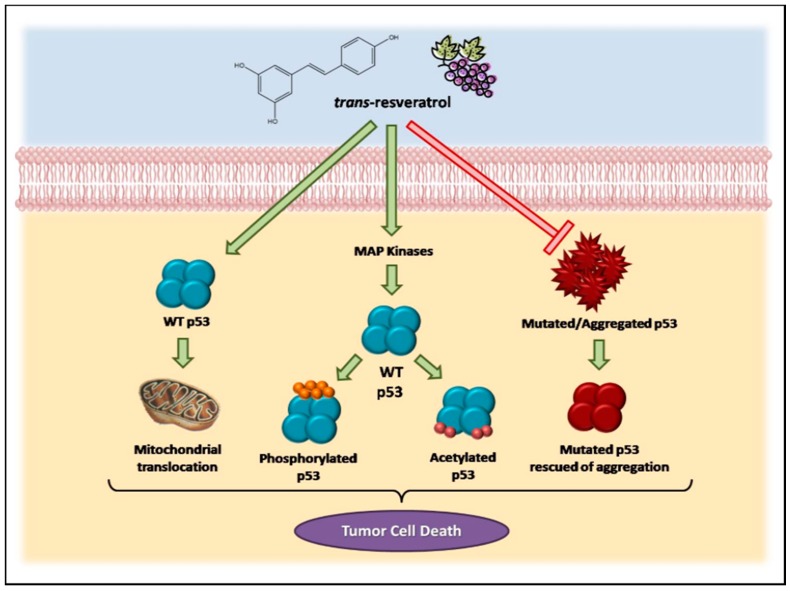
The involvement of the p53 pathway in the effects triggered by resveratrol in cancer cells.

All together, these data demonstrate the involvement of the p53 pathway in the effects triggered by resveratrol in cancer cells, as summarized in Figure 1.

## 4. Bioavailability of Resveratrol

The low bioavailability and extensive metabolism of resveratrol are a constant source of concern regarding whether the concentrations used for most in vitro studies are even relevant in vivo [108]. One of the biggest challenges for resveratrol in therapy is its poor bioavailability. Due to its rapid phase II metabolism in the liver and intestine [108,109], the bioavailability of ingested and intravenous doses of resveratrol is unable to achieve pharmacologically active concentrations in plasma [110], but the enterohepatic recirculation may contribute to a delayed elimination of the drug from the body and introduce a prolonged effect. By its binding to plasmatic proteins, resveratrol also exhibits a prolonged effect [11].

It is clear that only a small fraction of ingested resveratrol reaches the body as the parent compound. Furthermore, the amount of resveratrol ingested from dietary sources, such as red wine and juices, among others, would very rarely exceed 5 mg, resulting in plasma levels that are either not detectable or are orders of magnitude below the micromolar concentrations employed in vitro. The administration of approximately 25 mg of resveratrol results in plasma concentrations of the free form ranging from 1 to 5 ng/mL [111]; the administration of higher doses (up to 5 g) produced concentrations of free resveratrol up to approximately 500 ng/mL, or just over 2 µM [112].

The actual plasma level and absorption kinetics are highly dependent on other compounds present in the dietary matrices. Therefore, a higher degree of bioavailability has been observed when resveratrol is obtained through wine and/or ingestion via the diet as a single pure compound [113]. Pignatelli et al. [114] observed a mean increase of 1 µM resveratrol in the plasma upon ingestion of 300 mL of red wine a day for 15 days. The actual resveratrol level in the wine was not known in these experiments, but assuming a mean level of resveratrol in red wine of 8.2 µM indicates a relatively high bioavailability [115]. Similarly, a comparison of the metabolic profile of resveratrol as a component of red wine and of grape extract revealed reduced resveratrol absorption and an extended presence in the gut when resveratrol was a component of grape extract [116].

Approximately 20–30% of resveratrol is not recovered in urine or feces [108], causing one to question whether resveratrol is still present in cells throughout the body. It is possible that resveratrol is associated with lipid compartments and released slowly [117].

Some studies have emphasized that other polyphenols present in red wine, such as quercetin, catechin, and gallic acid, could function as potential chemopreventive agents [118,119,120]. From this perspective, the research group led by Dr. Latruffe has previously shown that a mixture of polyphenolic extracts from grape vine shoots exhibits superior anti-proliferative activity in colon cancer cells to resveratrol alone due to a synergistic interaction between polyphenols [121,122].

The combination of resveratrol with various stilbenes did show synergism (with pterostilbene and polydatin) when analyzing the antioxidant as well as the cytostatic effects [123,124]. The combination of curcumin with resveratrol has been tested in various in vitro models, with the antioxidant capacity, cytostatic effects, and induction of apoptosis being evaluated [125]. Various flavonoids have been tested in combination with resveratrol, including chrysin, quercetin, catechin, genistein, and combinations of several flavonoids. The effects varied depending on the measured activity and combinations of the tested compounds. In general, the same response, but at lower concentrations, was most often observed by using the combined compounds [117]. The role of ethanol is also potentially important in improving the solubility of polyphenols and altering the cell membrane fluidity for cellular uptake, while a high level of ethanol could counteract the beneficial effects of polyphenols [122].

The metabolism of resveratrol by the human microbiota is another confusing factor in relation to the level of resveratrol and metabolites in the body. A changed metabolite profile was observed when using human fecal microbiota [126], which is why metabolism by the gastrointestinal microbiota is also relevant. Aires et al., 2013 [127] have provided significant new insights into the molecular mechanism of resveratrol, and their data support the notion that, despite low bioavailability in vivo, the biological effects of resveratrol could be mediated by its metabolites.

Modifications of resveratrol stability, chemical structure, and metabolism could alter its cellular and molecular targets and could be crucial for improving or decreasing the efficiency of its chemopreventive properties [25]. Interest in the bioproduction and chemical synthesis of stilbenes has also emerged to identify highly active molecules that could be used for medical applications, especially for cell proliferation inhibition [128]. Modification of the hydroxylation and methoxylation patterns of resveratrol had inhibitory effects on the human colorectal tumor SW480 cell line and no effects on non-tumor cells (IEC18 intestinal epithelial cells), demonstrating the selectivity of these molecules for cancer cells [129].

Cisplatin, carboplatin, and oxaliplatin are commonly used chemotherapy drugs that crosslink DNA in rapidly growing cells. In most experiments, an additive effect and sometimes a synergistic effect has been observed on the reduction of the cell viability of various cancer cell lines [130,131]. Similar effects were observed with DNA intercalating drugs such as doxorubicin and docetaxel [132], topoisomerase inhibitors (such as etoposide) [133], and nucleotide analogs (fluorouracil, fludarabine, cladribine, gemcitabine, clofarabine, and decitabine) [134]. Additionally, the combination of resveratrol with DNA-alkylating substances (cyclophosphamide, temozolomide, melphalan, and carmustine) resulted in a potentiation compared with the effect of the drug alone [135]. Reduced or counteracting effects of resveratrol have also been observed when resveratrol is combined with inhibitors of microtubules (vinblastine and paclitaxel), depending on the order of treatment [130]. Similarly, the cytotoxic effect of the proteasome inhibitor MG132 is reduced by resveratrol [51]. In several cases, a synergistic effect has been postulated.

Resveratrol may act in an additive or synergistic manner with other polyphenols and may influence the metabolism or activity of other drugs. The synergism of various polyphenols with resveratrol has been observed experimentally [123,136,137,138] and underlies the effects of many nutraceutical formulations.

This challenge, the use of natural or synthetic analogs with improved bioavailability or more potency than resveratrol, as well as the combinations of drugs that provide a synergistic effect or improved bioavailability are promising strategies, as in the case of quercetin and other flavonoids [139]. This last method is very attractive as an anticancer drug therapy because the combination of drugs might result in the use of lower doses of individual compounds, leading to better pharmacological action due to additive or synergistic effects, and less collateral effects on the organism [140,141].

Additionally, nanoencapsulated resveratrol has shown enhanced bioavailability relative to the parent compound [142,143]. Encapsulation and the use of alternative routes of uptake have also been explored [144,145].

## 5. Clinical Trials and Therapeutic Perspectives for the Use of Resveratrol

It is important to note that often there are discrepancies between the doses of resveratrol used in cells and levels obtained in vivo. For example, many studies showing an effect of resveratrol on signaling used concentrations ranging from 10 μM to 100 μM [146]. In contrast, a single 25 mg dose of resveratrol, corresponding to high red wine consumption, resulted in marginal levels of plasma resveratrol in human subjects, and a 5 g dose produced a transient peak of only 2.4 μM [147]. When lower concentrations corresponding to plasma levels are used on cells, the outcomes are variable, and often, no effects are detected [148]. Almeida et al. [111] showed that repeated dosing can increase the plasma half-life of resveratrol by more than two-fold.

The duration of the trials varies from acute exposure [149,150], to a few days [7,151,152,153] of exposure [154,155], to up to one year [156]. In most trials, the subjects are exposed to resveratrol in time frames of 1–3 months. The relatively short duration of the trials is a challenge because they permit analysis of therapeutic but not preventive potential. Without taking the practical and economical aspects of such studies into consideration, a trial to show a preventive effect of resveratrol should be performed for a minimum of one year. It is obvious that such trials are expensive and not easily funded, but they will be necessary to obtain relevant information regarding the preventive/therapeutic potential of resveratrol [148].

Notably, the resveratrol doses available in supplements and used in many clinical trials are 2–3 orders of magnitude beyond what can be obtained from the diet [157]. It is difficult to estimate normal human consumption of resveratrol because the intake of red wine differs greatly in the population and the content of resveratrol varies (mean, 1.9 ± 1.7 mg/L), but the dose may reach 4 mg/person/day [115]. A single dose of resveratrol results in plasma levels of 2–18 µµM [149]. Ingestion of 3 g daily in obese individuals for eight weeks caused rapid and extensive conjugation of resveratrol [158].

Resveratrol trials in humans after single [108,147] or multiple daily doses of up to 600 mg/day administered over two or three days [111,159] showed that resveratrol is safe under the tested conditions and that the main metabolites found in the circulation are R3S, R4G, and R3G, with particularly high levels in the case of the sulfo-conjugate [160]. A few phase 1 clinical trials focusing on the pharmacokinetics of resveratrol were published before 2010, but since then, the number of clinical trials exploring the biological effects of resveratrol has increased significantly.

In recent years, resveratrol has been shown to possess a fascinating spectrum of pharmacologic properties that could be useful in human medicine [157,161]. To search for clinical trials with resveratrol to write this review, the clinicaltrials.gov site was accessed in April 2017, and 129 studies were identified. After excluding non-cancer studies, only 20 trials remained. Oral resveratrol is used as a pure compound or in resveratrol-rich products (grapes, grape juice or a mixture of supplements), and the doses and durations of the interventions differed, as shown in Table S1. The following are the 20 clinical trials that were identified.

**Clinical trial 1:** “**Resveratrol for Patients with Colon Cancer**”. A trial conducted by Nguyen et al. [162], with biomarker endpoints evaluating the expression of multiple components and target genes of the Wnt pathways, represents the first reported clinical trial of resveratrol in patients with cancer. The first two patients receiving resveratrol will be treated at a dose of 20 mg/day, the third and fourth patients at a dose of 80 mg/day, and the fifth and sixth patients with a dose of 160 mg/day. All patients receiving grape extract will receive 125 mg/day that will have to be mixed with one 8 oz glass of water. There will be no dose adjustments. If a patient has any side effects which are attributed to the resveratrol, it will be discontinued. The conclusion of the study was that grape powder containing low doses of resveratrol in combination with other bioactive components suppressed the expression of Wnt target genes, cyclin D1 and axin in normal colonic mucosa, suggesting that Wnt pathway inhibition might contribute to resveratrol-mediated colon cancer prevention.

**Clinical trial 2:** “**A Biological Study of Resveratrol’s Effects o**n Notch-1 Signaling in Subjects with Low Grade Gastrointestinal Tumors” is the work of Emily R. Winslow from the University of Wisconsin, Madison. Resveratrol has been shown to activate Notch-1, the signaling of which prevents tumor cell growth. This trial examines the effects of resveratrol and Notch-1 on neuroendocrine tumor tissue and the tolerance of people with neuroendocrine tumors who take resveratrol for up to three months (5 g per day orally administered in two divided doses of 2.5 g each with minimal dose-limiting toxicities according to the National Cancer Institute (NCI) Common Toxicity Criteria). The levels of tumor markers (e.g., chromogranin, 5-HIAA, gastrin, and others) pretreatment will be compared with the post-treatment levels (collected every three months) as a measure of the tumor response. In addition, serial axial imaging will be used to document tumor response rates according to standard Response Evaluation Criteria in Solid Tumors (RECIST).

**Clinical trial 3:** “**Resveratrol and Human Hepatocyte Function in Cancer**” is a trial coordinated by Dr. Brian G. Harbrecht from the University of Louisville. Resveratrol has shown a beneficial effect on the cellular function of normal and cancer liver cells in samples of liver tissue collected during elective liver surgery. Outcomes based on three measures will test the hypothesis that resveratrol used as a nutritional supplement (1 g pill daily) for 10 days before surgery will have the following effects: (1) improve metabolic function in liver cells, as assessed by the expression of signaling proteins, such as Akt, p38, MAPK, AMPK and PEPCK; (2) reduce cellular growth and proliferation of cancer cells, based on the expression of genes and proteins such as cyclin and p53 and the apoptosis proteins Bcl-2 and Bcl-xL; and (3) decrease inflammation in the liver, as determined by evaluating different levels of genes and proteins for nitric oxide synthase, cytokines such as interleukin-6, and nuclear factor-kappa B signaling proteins. Unfortunately, this trial was withdrawn prior to enrollment.

**Clinical trial 4:** “**Phase I Biomarker Study of Dietary Grape-Derived Low Dose Resveratrol for Colon Cancer Prevention**” from the University of California, Irvine, was conducted by Randall F. Holcombe. The purpose of this study was to determine the minimum amount of resveratrol-rich fresh red grapes needed to exhibit signs of colon cancer prevention. The grape-supplemented diet provided a low dose of resveratrol in conjunction with other potentially active components contained within the grapes. Colon tissue was obtained by limited flexible sigmoidoscopy before and after ingestion of the red grape-containing diet. Different dosages of grapes (1 or 2/3 or 1/3 lb/day fresh red grapes) were utilized. This study showed the effects of dietary grape-derived low-dose resveratrol on biomarkers related to the Wnt pathway and provided critical information regarding the utility of this nutritional approach for the prevention of colon cancer.

**Clinical trial 5.** The phase 1 trial, “**Resveratrol in Treating Patients with Colorectal Cancer That Can Be Removed by Surgery**”, conducted by the research group of Dr. Brenner D., coordinated by the National Cancer Institute, addressed the side effects and the optimal dose of resveratrol for the treatment of patients with colorectal cancer that can be removed by surgery. Resveratrol was offered from eight days before colectomy and potentially halted tumor cell growth by blocking M-1G adducts and levels of cyclooxygenase-2 protein/Ki67.

**Clinical trial 6** is a phase 1 trial, “**UMCC 2003-064 Resveratrol in Preventing Cancer in Healthy Participants (IRB 2004-535**)”, led by Dean Brenner from the University of Michigan Cancer Center to study the side effects and optimal dose of resveratrol for the prevention of cancer in healthy participants. The concentration of resveratrol and its metabolites were analyzed in the plasma, urine, and feces. Beginning five days before study drug administration, participants were placed on a controlled diet (avoiding all resveratrol-containing food or drink) for washout. The participants received oral resveratrol once on Day 1 and were followed at two and seven days. To assess safety, pharmacokinetics and the insulin-like growth factor axis after repeated doses of resveratrol, Brown and colleagues [4] from Dr. Dean Brenner research group recruited forty volunteers to ingest resveratrol at dosages of 0.5 g, 1.0 g, 2.5 g or 5.0 g daily for 29 days and only sixteen participants are treated at the maximum tolerable dose. The higher doses (2.5 g and 5.0 g) generated micromolar concentrations of the parent and substantially higher levels of glucuronide and sulfate conjugates in the plasma and caused gastrointestinal symptoms of mild to moderate severity. The observed decrease in circulating IGF-1 and IGFBP-3 may contribute to cancer chemopreventive activity.

**Clinical trial 7:** “**A Clinical Study to Assess the Safety, Pharmacokinetics, and Pharmacodynamics of SRT501 in Subjects with Colorectal Cancer and Hepatic Metastases**” is a randomized, double-blind, placebo-controlled, inpatient/outpatient study. Micronized resveratrol (SRT501) was given at a dosage of 5 g daily for 14 days to patients with colorectal cancer with hepatic metastases scheduled to undergo hepatectomy. Micronization allows increased resveratrol absorption, thus increasing its availability, and SRT501 was well tolerated. Mean plasma resveratrol levels following a single dose of SRT501 were 1.9 ± 1.4 ng/mL, exceeding those published for equivalent doses of non-micronized resveratrol by 2- to 3.5-fold [4,147]. Resveratrol was detectable in hepatic tissue following SRT501 administration. Cleaved caspase-3, a marker of apoptosis, was significantly increased by 39% in malignant hepatic tissue following SRT501 treatment compared with tissue from placebo-treated patients [163].

**Clinical trial 8:** “**Resveratrol in Healthy Adult Participants**” is led by the principal investigator, Dr. Hsiao-Hui (Sherry) Chow, from Arizona Cancer Center, Tucson. Samples of blood and urine from healthy adult participants who are taking resveratrol once daily for four weeks are being studied. This phase 1 trial is studying the side effects of resveratrol and assessing its effects on healthy adult participants. This trial will compare the CYP enzyme activities from baseline to the end of the resveratrol intervention. CYP1A2, 2D6, 2C9, and 3A4 activity will be assessed according to the plasma paraxanthine/caffeine ratio, urinary dextromethorphan/dextrorphan ratio, urinary losartan/losartan metabolite ratio, and area under the plasma buspirone concentration-time curve, respectively. Additionally, GST activity and GST-pi level in blood lymphocytes and serum bilirubin levels will be used to assess phase II enzyme activity. Finally, a safety evaluation using the NCI Common Terminology Criteria for Adverse Events (CTCAE) version 3.0 will be conducted, and any adverse events will be described.

**Clinical trial 9:** “**Resveratrol in Postmenopausal Women with High Body Mass Index**” is a pilot phase 1 trial examining resveratrol in postmenopausal women with a high body mass index to determine the ability of resveratrol to modulate circulating sex steroid hormones and estrogen metabolites to evaluate its potential for breast cancer prevention. Forty subjects initiated the resveratrol intervention (1 g daily for 12 weeks), and six withdrew early due to adverse events. The resveratrol intervention did not result in significant changes in serum concentrations of estradiol, estrone, or testosterone, but it led to, on average, a 10% increase in the concentrations of sex steroid hormone binding globulin (SHBG). It also resulted in, on average, a 73% increase in urinary 2-hydroxyestrone (2-OHE1) levels, leading to a favorable change in the urinary 2-OHE1/16α-OHE1 ratio. One participant had a symptomatic grade 4 elevation of liver enzymes at the end of the study intervention. Two subjects had grade 3 skin rashes. The most common adverse events were diarrhea and increased total cholesterol, which were reported in 30% and 27.5% of the subjects, respectively. The authors concluded that among overweight and obese postmenopausal women, a 1 g dose of resveratrol daily has favorable effects on estrogen metabolism and SHBG [164]. Clinical trial reports indicate that the research group of Dr. Chow intends to evaluate the effects of resveratrol on serum levels of C-peptide, serum C-reactive protein (CRP), and adipocytokine expression and secretion as measured by serum leptin and adiponectin. Urinary 8-isoprostaglandin F2 alpha (8-iso-PGF2 alpha) and 8-hydroxydeoxyguanosine (8OHdG) will be assessed to evaluate oxidative stress, and the safety of resveratrol intervention will be measured by the reported adverse events, complete blood count with differential (CBC/diff), comprehensive metabolic panel (CMP), and lipid profile.

**Clinical trial 10:** “**Resveratrol with or without Piperine to Enhance Plasma Levels of Resveratrol**”. There is some evidence that resveratrol in combination with piperine (an alkaloid found in pepper) is more effective for fighting cancer, and therefore, the purpose of this study is to determine whether this combination is more effective than resveratrol alone. Because the investigators do not know the dose of piperine to use in combination with resveratrol, two different doses of piperine will be studied. Twenty-four participants, comprising equal numbers of males and females, will receive, for 30 days, a single dose of resveratrol (2.5 g) without piperine, resveratrol (2.5 g) with piperine at 5 mg, or resveratrol (2.5 g) with piperine at 25 mg. Blood levels of resveratrol and piperine will be measured, and adverse events and side effects will be analyzed. This trial is led by Howard H Bailey from the University of Wisconsin, Madison and has collaborators at the National Institutes of Health (NIH) and the NCI.

**Clinical trial 11: “Pilot Study of Resveratrol in Older Adults with Impaired Glucose Tolerance”**. This proposed pilot study will examine resveratrol treatment (500 mg capsules, three capsules (1500 mg) orally twice a day for six weeks) in older adults with impaired glucose tolerance (IGT) to explore its effects on post-meal blood glucose metabolism.

**Clinical trial 12: “Resveratrol and Cardiovascular Health in the Elderly”**. The objectives of this study are to test the effects of different doses of resveratrol (75 mg or 150 mg, 2×/day, orally) on heart and blood vessel health. This phase 1/2 trial of resVida (an oral preparation of resveratrol) in 90 overweight/obese people over the age of 50 (30 in each group) will be conducted for 12 months.

**Clinical trial 13: “Resveratrol and the Metabolic Syndrome”**. The investigators propose to validate that resveratrol, administered to subjects with the metabolic syndrome under controlled conditions of weight stability, common diet, and strict compliance with the study drug, will improve the symptoms of the metabolic syndrome, thereby decreasing the chance of developing diabetes or heart disease.

**Clinical trial 14:** “**Dietary Intervention in Follicular Lymphoma (KLYMF)**” from Oslo University Hospital by Dr. Harald Holte, Jr. The recruitment status of this study is unknown. The completion date has passed, and the status has not been verified for more than two years. This open study projecting to include 45 patients seeks to perform a dietary intervention for 16 weeks and compare the apoptosis rate, proliferation rate and immune cell infiltrate before and after the intervention period. A dietary intervention study in patients with follicular lymphoma (FL) Stage III/IV consists of omega 3 fatty acids (eicosapentaenoic acid (EPA) and docosahexaenoic acid (DHA)) 1000 mg × 5 daily, selenium (L-Seleno methionine) 100 mcg × 2 daily, garlic extract (Allicin) 6 garlic pearls daily; and 100% pomegranate juice (ellagic acid), grape juice (resveratrol, quercetin), and green tea (epigallocatechin gallate), in a volume of two cups daily for 16 weeks.

**Clinical trial 15:** A phase 1 study for metastatic colorectal patients [163] did not report nephrotoxicity. As SRT501 is extensively metabolized, renal failure appeared to be specific to multiple myeloma patients [165] due to multiple causes, and hence all patients are at risk. **“A Clinical Study to Assess the Safety and Activity of SRT501 Alone or in Combination with Bortezomib in Patients with Multiple Myeloma”**. Twenty-four patients were enrolled in a phase 2 clinical trial of 5 g of SRT501 with and without bortezomib in patients with multiple myeloma who had relapsed or were refractory to at least one prior therapy. Disease stabilization by bortezomib may have prevented renal failure, whereas low efficacy of SRT501 with nausea and vomiting may have resulted in disease progression and dehydration, leading to renal failure. Renal toxicity was observed in five of the twenty-four patients. Renal failure is one of the specific clinical features of multiple myeloma and can be observed in nearly half of patients throughout the disease [166]. This study demonstrated an unacceptable safety profile and minimal efficacy in patients with relapsed/refractory multiple myeloma, highlighting the risks of novel drug development in such populations [167].

**Clinical trial 16: “Anti-inflammatory and Antioxidant Effects of Resveratrol on Healthy Adults**”, is a clinical trial phase 3 that investigated the hypothesis that resveratrol (500 mg) administered orally to healthy adult smokers induces a decrease in inflammatory and oxidative mediators characterizing the low-grade systemic inflammatory state and the oxidant-antioxidant imbalance of tobacco users. Markers of total antioxidant/oxidative stress (C-reactive protein, 4-hydroxynonenal, nitrotyrosine, endothelial nitric oxide synthase (eNOS)-polymorphism, superoxide dismutase (SOD2)-polymorphism, catalase-polymorphism, pentraxin 3, interleukin-6, tumor necrosis factor-α) will be analyzed at baseline and every 30 days for three months. This trial will be conducted by Dr. Simona Bo from the University of Turin, Italy.

**Clinical trial 17:** “**Study of Supplement’s Antioxidant Properties That Contains Natural Extracts**”. The possibility has been raised that the complex mixture of phytochemicals in foods may contribute to their protective effects. In this view, it is possible that multiple compounds act through complimentary or synergistic mechanisms to provide a greater biologic effect than can be achieved by any individual component/nutrient. To investigate this hypothesis, a double-blind, randomized, and placebo-controlled clinical trial was conducted by Dr. Elizabeth Fragopoulou, from Harokopio University to investigate the effects of a multi-micronutrient supplement against oxidative stress (isoprostane, DNA/RNA damage, protein carbonyl levels, oxLDL, TBARS, resistance to serum oxidation, antioxidant enzymes) in apparently healthy adults for four or eight weeks. The supplement contained the following per 80 mL (a daily dose): *Aloe barbadensis* miller gel (USA/Mexico 36%); grape juice, P*olygonum cus*p*idatum* extract (containing 10% resveratrol); green tea extract; 1.1 mg of vitamin B1 (100% RDA); 2.5 µg of vitamin B12 (100% RDA); 12 mg of vitamin E (α-ΤΕ) (100% RDA); coenzyme Q10; 200 µg of folic acid (100% RDA); ascorbic acid; 27.5 µg of selenium (100% RDA); and 4.2 mg of iron (100% RDA).

**Clinical trial 18:** “**Effects of Micronized Trans-resveratrol Treatment on Polycystic Ovary Syndrome (PCOS) Patients**”. The purpose of this study, conducted by Dr. Beata Banaszewska from Poznan University of Medical Sciences, Poland with the University of California as a collaborator, is to determine whether three months of therapy with micronized trans-resveratrol (500 mg) can improve clinical (excessive hair, menstrual cycle), endocrine (androgens) and metabolic (lipids, markers of systemic inflammation) profiles in women with PCOS.

**Clinical trial 19:** Resveratrol is able to potentiate simvastatin-induced inhibition of cell proliferation in a concentration-dependent manner and inhibit the mevalonate pathway, suggesting a novel mechanism of action of resveratrol and underscoring the potential translational/clinical relevance of the interaction of this microcomponent with simvastatin [150]. The phase 4 clinical trial “**Effects of Simvastatin and Micronized Trans-resveratrol Treatment on Polycystic Ovary Syndrome (PCOS) Patients”** is being conducted by Beata Banaszewska, Poznan University of Medical Sciences, Poland. This study is designed to evaluate the endocrine and metabolic effects of simvastatin (20 mg daily) and micronized trans-resveratrol (500 mg daily) on PCOS. Evaluations are performed at baseline and repeated after three and six months of treatment, and the main outcome is a change in serum total testosterone and fasting insulin levels.

**Clinical trial 20: “Evaluation of Oral Lipid Ingestion in Relation to Ovarian Androgen Secretion in Polycystic Ovary Syndrome (PCOS) (ELI-ROAS)”**. The purpose of this phase 1 trial is to determine the relationship between lipid-induced inflammation and ovarian androgen secretion in women with PCOS, and to examine the effect of the drug salsalate (2 g twice daily) and P*olygonum cus*p*idatum* extract (PCE) (200 mg containing 20% of resveratrol twice daily) for 12 weeks on lipid-induced inflammation, ovarian androgen secretion, body composition and ovulation in a subset of normal weight women with PCOS. The principal investigator, Dr. Frank González, Director of the Division of Reproductive Endocrinology and Infertility, from Indiana University, hypothesizes that in women with PCOS, HCG administration will stimulate an exaggerated ovarian androgen response, dairy cream ingestion will stimulate white blood cells to generate an inflammatory response and that there is a relationship between HCG-stimulated ovarian androgen secretion and the inflammatory response to dairy cream ingestion regardless of body fat status. Thirty (30) women with PCOS (10 normal weight with normal abdominal adiposity, 10 normal weight with increased abdominal adiposity and 10 obese) and 30 ovulatory control women (10 normal weight with normal abdominal adiposity, 10 normal weight with increased abdominal adiposity and 10 obese) will participate over a three-year period. The investigator also hypothesizes that both salsalate and PCE administration for 12 weeks will attenuate the ovarian androgen response to HCG administration and the inflammatory response to dairy cream ingestion, reduce abdominal adiposity, increase insulin sensitivity and induce ovulation in normal weight women with PCOS. In a subset of 16 women with PCOS, eight will receive salsalate (four normal weight with normal abdominal adiposity and four normal weight with increased abdominal adiposity) and eight will receive PCE (four normal weight with normal abdominal adiposity and four normal weight with increased abdominal adiposity) over a three-year period. This pilot project will help determine the feasibility of conducting a larger double-blind, randomized trial in women with PCOS to further test the latter hypothesis.

**Figure 2:**
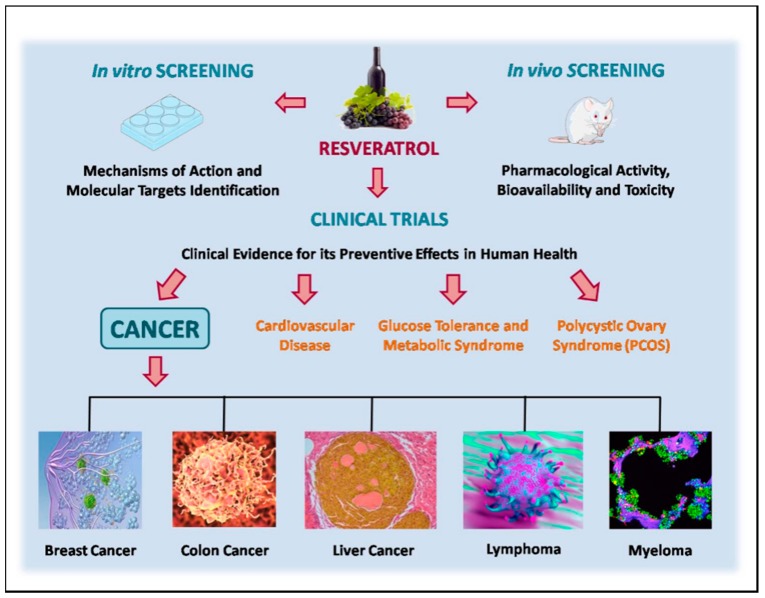
In vitro, in vivo and clinical evidence for the preventive effects of resveratrol in human health.

A summary of clinical evidence for the preventive effects of resveratrol in human health, especially in cancer, is presented in Figure 2.

Considering the twenty studies shown above, only ten (50% of total) were completed. Most of them are phase 1 clinical trials with the aim to look at doses and side effects. Eight of the clinical studies are associated to different types of cancer, showing that until now resveratrol is associated to preventive effects. We further suggest that the quality of clinical trials in future should be improved through an increased number of participants, appropriate study designs, new formulations and/or routes of administration, and biomarkers relevant to healthy participants. Unfortunately, at this moment, the preventive and therapeutic effects of resveratrol in humans are still only supported by in vitro and in vivo model organism studies.

## 6. Adverse Effects

Based on animal studies, resveratrol is generally well tolerated, and very few short-term or acute exposure experiments in humans have been performed. When eight healthy subjects were exposed to 2 g of resveratrol twice daily for eight days, six of eight subjects had mild episodic diarrhea/loose stool, typically at the beginning of the eight-day treatment period, and one of the subjects developed a temporary rash and headache [168]. In a double-blinded, randomized, placebo-controlled study, up to 975 mg/day were given to healthy volunteers, among which two adult subjects (male and female) in each group were subjected to 25 mg, 50 mg, 100 or 150 mg six times/day for a total of two days. Adverse effects were mild in severity and similar among all groups. Repeated administration of resveratrol was well tolerated but produced relatively low plasma concentrations of resveratrol, despite the high doses and short dosing interval used [111]. Exposure of up to 270 mg resveratrol to nineteen volunteers for one week did not cause any discomfort [150].

According to Elliott et al. [169], healthy volunteers tolerated resveratrol well in a seven-day exposure study, but experimental details were not provided, making evaluation of the results challenging. The same article describes very briefly a study that included daily exposure to 2.5 g or 5 g resveratrol for 28 days. The authors reported that adverse events were generally mild in nature and reversible, but no experimental details were provided, precluding a closer evaluation [169]. Twenty colon cancer patients who received 0.5 g or 1.0 g resveratrol daily for eight days before surgical resection showed good tolerance [154].

## 7. Recommendations

The first international conference on resveratrol and health, Resveratrol 2010, was held in September 2010 in Denmark, with the purpose of assessing the current state of knowledge in the field and making recommendations for human use and future studies of resveratrol. A clear theme of the conference, which was attended by all of the authors, was that multiple mechanisms are likely to contribute to the beneficial effects of resveratrol, making it difficult to agree on a specific dose, biomarker, or outcome that can define the molecule [157,170].

The second international conference on resveratrol and health, Resveratrol 2012, occurred at the University of Leicester in England and concluded that the published evidence from human trials is not sufficiently strong to justify the recommendation of chronic resveratrol consumption by humans for any given indication. New animal data and recent short-term clinical trials are promising and indicate the need for further long-term human clinical trials, and the use of resveratrol is not an alternative to maintaining a healthy lifestyle.

During the third International Conference on Resveratrol and Health, Resveratrol 2014 (Hawaii), longer exposure times to resveratrol in new trials were recommended. To date, no real clinical trials exploring resveratrol in relation to cancer have been published [171]. At the fourth International Conference on Resveratrol and Health, Resveratrol 2016 (Taiwan), the highlights were focused on the interesting effects of resveratrol on hemorrhagic and septic shock models, its promising neuroprotective abilities and positive influence in psoriasis, and its role in bacterial shift.

## 8. Conclusions

This review describes recent evidence regarding resveratrol as a chemopreventive agent and a conceptual framework for a new approach to cancer prevention and therapeutics, which is in high demand. A broad-spectrum approach involves synergistic combinations of multiple low-toxicity agents as an association of microcomponents (e.g., resveratrol plus another bioactive compound) or with chemotherapeutic drugs that can collectively impact many pathways that are known to be important for carcinogenic processes, in addition to angiogenesis and metastases. The potential targets of the effect of resveratrol were presented, and p53 as an important protein involved in carcinogenesis could represent a new focus for exploration in translational studies.

## Supplementary Materials

The following are available online: Table S1. Synthesis of clinical trials with resveratrol and cancer presented in the site of www.clinicaltrials.gov (accessed in April 2017).
