# Supplementary material for: Cancer Chemoprevention by Resveratrol: The p53 Tumor Suppressor Protein as a Promising Molecular Target
Source: Molecules. 2017 Jun 18;22(6):1014. doi: 10.3390/molecules22061014 (PMC6152653; doi:10.3390/molecules22061014)
Supplement: Supplementary file 1 [file molecules-22-01014-s001.pdf]

Table 1: Synthesis of clinical trials with resveratrol and cancer presented in the site of www.clinicaltrials.gov (accessed in April 2017).

Clinical Trials.gov identifier	Number/ Year of inclusion	Status	Phase	Purpose/condition	Study type	Dose / duration
NCT002556334	1 / 2005	Completed	Phase 1	To define the actions of resveratrol on the Wnt signaling pathway in a clinical trial in which patients with colon cancer received treatment with resveratrol, and correlative laboratory studies examined its effects directly on **colon cancer** and normal colonic mucosa	Intervention model: single group<br><br>Assignment masking: open label<br><br>Primary purpose: treatment	**Plant-derived resveratrol tablets** at a dose of **20** or **80 mg day** and **grape powder (GP)** dissolved in water and taken orally at a dose of **80** or **120** g/day.<br>Treatment will be approximately **14 days** before standard of care surgical resection of tumor
NCT01476592	2 / 2011	Active, not recruiting	Phase 1	To examine the effects of resveratrol and Notch-1 on **neuroendocrine tumor** tissue and to examine how people with neuroendocrine tumors who take resveratrol for up to three months tolerate the product.	Intervention model: single group<br><br>Assignment masking: open label<br><br>Primary purpose: treatment	**Resveratrol 5 g/day** orally, in two divided doses of 2.5 g each without a break in therapy for a total of three cycles.<br>Treatment will be for **3 months.**
NCT02261844	3 / 2014	This study has been withdrawn prior to	Phase 1<br>Phase 2	The purpose of this study is to determine if resveratrol, a nutritional supplement, shows a beneficial effect in the cellular	Allocation: randomized<br><br>Intervention	**Resveratrol 1g/ daily for 10 days**

		enrollment		function of normal liver cells and diseased **liver cells (cancer cells)** in samples of liver tissue taken during elective liver surgery.	model: parallel<br><br>Assignment masking: Participant<br><br>Primary purpose: basic science	
NCT00578396	4 / 2007	The recruitment status of this study is unknown. The completion date has passed and the status has not been verified in more than two years.	Phase 1	The purpose of this study is to determine the minimum amount of resveratrol-rich fresh red grapes needed to exhibit such signs of **prevention**.	Allocation: randomized<br><br>Intervention model: parallel<br><br>Assignment masking: open label<br><br>Primary purpose: prevention	Dietary supplement: **grapes** (1 lb/day fresh red grapes, dose level 1)<br><br>1 pound of **seedless red grapes** (2/3 lb/day **fresh red grapes**, dose level 2)<br><br>1/3 lb/day **fresh red grapes**
NCT00433576	5 / 2007	Completed	Phase 1	This phase I trial is studying the side effects and best dose of resveratrol in treating patients with **colorectal cancer** that can be removed by surgery.	Allocation: non-randomized<br><br>Intervention model: single group	**Resveratrol** given orally **1g for 8 or 9 days**

					Assignment masking: open label<br><br>Primary purpose: treatment	
NCT00098969	6 / 2004	Completed	Phase 1	This phase I trial is studying the side effects and best dose of resveratrol in **preventing cancer** in healthy participants.	Masking: open label<br><br>Primary purpose: prevention	16 participants receive escalating doses of **resveratrol** (**from 0.5 to 5.0 g daily**) until the maximum tolerated dose (MTD) Participants are followed at **2 and 7 days**.
NCT00920803	7 / 2009	Completed	Phase 1	The primary purpose of this study is to determine the safety and tolerability of **SRT501** in subjects with **colorectal cancer and hepatic metastases**.	Allocation: randomized<br><br>Intervention model: parallel<br><br>Assignment masking: double blind<br><br>Primary purpose: basic science	SRT501 was supplied in clinical kits as a powder which was reconstituted with vehicle and water into a liquid suspension. **SRT501 (5 g)** was administered orally, once daily for **14 days**.

NCT00721877	8 / 2008	Completed	Phase 1	This phase I trial studied the side effects of **resveratrol** and the effects on drug and carcinogen metabolizing enzymes.	Intervention model: single group<br><br>Assignment masking: open label<br><br>Primary purpose: prevention	Participants receive **oral resveratrol once daily for 4 weeks**.
NCT01370889	9 / 2011	Completed	Phase 1	In postmenopausal women with high body mass index were determined the effects of **resveratrol** on circulating sex steroid hormones and estrogen metabolites to evaluate its potentials for **breast cancer prevention**	Intervention model: single group<br><br>Assignment masking: open label<br><br>Primary purpose: basic science	**Fourty subjects** initiated the **resveratrol** intervention (**1 g daily for 12 weeks**) with six withdrawn early due to adverse events
NCT01324089	10 / 2011	Completed	**Phase 1**	The purpose of this study is to see if **resveratrol** in combination with piperine is more effective than taking **resveratrol** alone. Since investigators don't know what dose of piperine to use in combination with **resveratrol**, two different doses of piperine were studied.	Allocation: randomized<br><br>Intervention model: parallel<br><br>Assignment masking: double blind	Twenty-four participants, equal numbers of males and females, received a single dose of **resveratrol** (**2.5 grams**) without piperine, **resveratrol** (2.5 grams) with piperine (5 mg), or **resveratrol** (**2.5 grams**) with piperine (25 mg).

					Primary purpose: prevention	
NCT01375959	11 / 2011	The recruitment status of this study is unknown. The completion date has passed and the status has not been verified in more than two years.	Phase 1	**Resveratrol** has many potential benefits, including prolonging lifespan, **preventing cancer** and heart disease and normalization of glucose metabolism. Although use of this agent shows great promise in the **treatment and/or prevention of diabetes.**	Allocation: randomized intervention<br><br>Model: crossover assignment<br><br>Masking: double blind<br><br>Primary purpose: treatment	500 mg capsules, 3 capsules (**1500 mg of resveratrol**) orally twice a day for 6 weeks
NCT01842399	12 / 2013	This study is currently recruiting participants	Phase 1<br>Phase 2	**Resveratrol** has many potential benefits, including prolonging lifespan, **preventing cancer** and heart disease and normalization of glucose metabolism. The purpose of this trial is to test the effects of different dose levels of resveratrol on **heart and blood vessel health**.	Allocation: randomized<br><br>Intervention model: parallel assignment<br><br>Masking: double blind<br><br>Primary purpose: prevention	**Resveratrol (75 mg or 150mg, 2x/day, orally) for 12 months**

NCT01714102	13 / 2012	This study is ongoing, but not recruiting participants	Phase 2	The underlying causes of metabolic syndrome are obesity, being overweight, physical inactivity and genetic factors. **Resveratrol** can decrease the chance of developing **diabetes, cancer and/or heart disease**.	Allocation: randomized<br><br>Intervention model: parallel<br><br>Assignment masking: double blind<br><br>Primary purpose: treatment	**Resveratrol (1 g) for 30 days**
NCT00455416	14 / 2007	The recruitment status of this study is unknown. The completion date has passed and the status has not been verified in more than two years.	Phase 2	A dietary intervention study in patients with Follicular Lymphoma stage III/IV to assess the ability of **several dietary factors** to induce apoptosis, inhibit cell proliferation and modulate **tumor** cell infiltrate *in vivo*.	Allocation: non-randomized<br><br>Intervention model: single group<br><br>Assignment masking: open Label<br><br>Primary purpose: treatment	The dietary intervention is composed of omega 3 fatty acids (EPA (eicosapentaenoic acid) and DHA (docosahexaenoic acid)) 1000 mg x 5 daily, Selenium (L-Seleno methionine), 100 mcg x 2 daily, Garlic extract (Allicin), 6 garlic pearls daily; Pomegranate juice 100% (ellagic acid), **Grape juice (resveratrol, quercetin**), Green Tea (Epigallocathechin gallate), 2 cups daily for 16 weeks.
NCT00920556	15 / 2009	This study has been terminated.	Phase 2	The primary purpose of this study is to determine the safety and tolerability of	Intervention model: single group	**SRT501 (5 g)** will be supplied in clinical kits as a

				**SRT501** with or without bortezomib administration, when administered once daily in 21 day cycles, in male and female subjects with **multiple myeloma**.	Assignment masking: open label<br><br>Primary purpose: treatment	powder which will be reconstituted with vehicle and water into a liquid suspension. **SRT501** will be administered **orally as a liquid suspension for 20 consecutive days in each 21 day cycle.**<br>Bortezomib (1.3 mg/m^2) will be given as an intravenous solution in a 3-5 second push on Day 1, 4, 8 and 11 in every 21 day cycle.
NCT01492114	16 / 2011	Completed	Phase 3	This research will investigate the hypothesis that **resveratrol** when given orally to healthy adult smokers induces a decrease in the inflammatory and oxidative mediators which characterize the low-grade systemic inflammatory state and the oxidants-antioxidants imbalance of **tobacco users**.	Allocation: randomized<br><br>Intervention model: crossover<br><br>Assignment masking: double blind<br><br>Primary purpose: prevention	**Resveratrol (500 mg**) first was submitted to: **30 days** of treatment, one tablet/day in the morning at fasting; then to 30 days of wash-out (no supplementation), and then to 30 days of treatment with placebo (one tablet/day in the morning at fasting).

NCT02837107	17 / 2016	This study is ongoing, but not recruiting participants.	Phase 1	This clinical trial was conducted in order to investigate the effects of a multi-micronutrient supplement against oxidative stress in apparently **healthy adults**.	Allocation: randomized<br><br>Intervention model: parallel<br><br>Assignment masking: double blind<br><br>Primary purpose: prevention	Dietary supplement: 80mL Mind Master* / day for 8 weeks
NCT01720459	18 / 2012	The recruitment status of this study is unknown. The completion date has passed and the status has not been verified in more than two years.	Phase 1	Effects of micronized trans-resveratrol treatment on clinical, endocrine, metabolic and biochemical parameters of women with **polycystic ovary syndrome**	Allocation: randomized<br><br>Intervention Model: parallel<br><br>Assignment masking: single blind<br><br>Primary purpose: treatment	**Micronized trans-resveratrol (500 mg) for 3 months**
NCT02766803	19 / 2016	This study is currently	Phase 4	This study is designed to evaluate the endocrine and metabolic effects of	Allocation: randomized	Drug: Simvastatin (20 mg) and **micronized trans-**

		recruiting participants		simvastatin and resveratrol on **polycystic ovary syndrome**	Intervention model: single group<br><br>Assignment masking: double blind<br><br>Primary purpose: treatment	**resveratrol (500 mg)**<br>**Maximum 6 months of treatment**
NCT01489319	20 / 2011	This study has been terminated.	Phase 1	Effect of salsalate and ***Polygonum cuspidatum*** **extract (PCE)** containing **resveratrol** on lipid-induced inflammation, ovarian androgen secretion, body composition and ovulation in a subset of normal weight women with **polycystic ovary syndrome**	Allocation: non-randomized<br><br>Intervention model: parallel<br><br>Assignment masking: no masking<br><br>Primary purpose: basic science	Drug: Salsalate<br>4 out of 10 subjects will receive salsalate 2.0 gm twice a day for 12 weeks; 4 out of 10 subjects will receive ***Polygonum cuspidatum*** **extract (PCE) 200 mg containing 20% resveratrol** twice a day for **12 weeks**.

*The supplement contained per 80ml, *Aloe barbadensis* miller gel (USA/Mexico 36%), grape juice, *Polygonum cuspidatum* extract (that contain 10% resveratrol), green tea extract, 1.1 mg vitamin B1 (100% RDA), 2.5 μg vitamin B12 (100% RDA), 12 mg vitamin E (α - TE) (100% RDA), coenzyme Q10, 200 μg folic acid (100% RDA), ascorbic acid, 27.5 μg selenium (100% RDA), 4.2 mg iron (100% RDA).
